# The Parent Trauma Response Questionnaire (PTRQ): development and preliminary validation

**DOI:** 10.1080/20008198.2018.1478583

**Published:** 2018-06-20

**Authors:** Victoria Williamson, Rachel M. Hiller, Richard Meiser-Stedman, Cathy Creswell, Tim Dalgleish, Pasco Fearon, Ben Goodall, Anna McKinnon, Patrick Smith, Isobel Wright, Sarah L. Halligan

**Affiliations:** a Department of Psychology, University of Bath, Bath, UK; b School of Psychology and Clinical Language Sciences, University of Reading, Reading, UK; c Department of Clinical Psychology, University of East Anglia, Norwich, UK; d Medical Research Council Cognition and Brain Sciences Unit and Cambridgeshire and Peterborough NHS Foundation Trust, Cambridge, UK; e Department of Clinical, Education and Health Psychology, University College London, London, UK; f Institute of Psychiatry, Psychology, and Neuroscience, King’s College London, London, UK

**Keywords:** Parent, child, adolescent, trauma, post-traumatic stress, questionnaire, Padre, niño, adolescente, trauma, postraumáticoestrés, cuestionario, 父母, 儿童, 青少年, 外伤, 创伤后压力, 调查问, • Parents are considered important for children’s post-trauma psychological outcomes.• The ability to assess their role in children’s post-trauma well-being has been hampered by a lack of validated trauma-specific parenting measures.• Drawing on five UK child trauma samples, we developed the Parent Trauma Response Questionnaire (PTRQ), a self-report measure of trauma-related appraisals and adaptive and maladaptive support styles.• We found preliminary evidence of validity and reliability of the PTRQ, including through association with child PTSD symptoms.

## Abstract

**Background**: Following a child’s experience of trauma, parental response is thought to play an important role in either facilitating or hindering their psychological adjustment. However, the ability to investigate the role of parenting responses in the post-trauma period has been hampered by a lack of valid and reliable measures.

**Objectives**: The aim of this study was to design, and provide a preliminary validation of, the Parent Trauma Response Questionnaire (PTRQ), a self-report measure of parental appraisals and support for children’s coping, in the aftermath of child trauma.

**Methods**: We administered an initial set of 78 items to 365 parents whose children, aged 2–19 years, had experienced a traumatic event. We conducted principal axis factoring and then assessed the validity of the reduced measure against a standardized general measure of parental overprotection and via the measure’s association with child post-trauma mental health.

**Results**: Factor analysis generated three factors assessing parental maladaptive appraisals: (i) permanent change/damage, (ii) preoccupation with child’s vulnerability, and (iii) self-blame. In addition, five factors were identified that assess parental support for child coping: (i) behavioural avoidance, (ii) cognitive avoidance, (iii) overprotection, (iv) maintaining pre-trauma routines, and (v) approach coping. Good validity was evidenced against the measure of parental overprotection and child post-traumatic stress symptoms. Good test–retest reliability of the measure was also demonstrated.

**Conclusions**: The PTRQ is a valid and reliable self-report assessment of parenting cognitions and coping in the aftermath of child trauma.

## RESUMEN

**Antecedentes**: Luego de una experiencia de trauma infantil, se piensa que la respuesta de los padres juega un papel importante, ya sea facilitando o dificultando su ajuste psicológico. Sin embargo, la capacidad de investigar el papel de las respuestas de los padres en el período posterior al trauma se ha visto obstaculizada por la falta de medidas válidas y confiables.

**Objetivos**: El objetivo de este estudio fue diseñar y proporcionar una validación preliminar del cuestionario de respuesta parental al trauma (PTRQ), una medida auto-aplicada de las apreciaciones de los padres y el apoyo para el afrontamiento de los niños, después del trauma infantil.

**Métodos**: Administramos un conjunto inicial de 78 ítems a 365 padres cuyos hijos, de entre 2 y 19 años, habían experimentado un evento traumático. Realizamos el eje principal y luego evaluamos la validez de la medida reducida frente a una medida general estandarizada de sobreprotección parental y mediante la asociación de la medida con la salud mental postraumática del niño.

**Resultados**: El análisis factorial generó tres factores que evalúan las evaluaciones desadaptativas de los padres: (i) cambio/daño permanente, (ii) preocupación por la vulnerabilidad del niño, y (iii) auto-culpa. Además, se identificaron cinco factores que evalúan el apoyo de los padres para el afrontamiento infantil: (i) evitación del comportamiento, (ii) evitación cognitiva, (iii) sobreprotección, (iv) mantenimiento de las rutinas previas al trauma y (v) enfoque de afrontamiento. Se evidenció una buena validez frente a la medida de la sobreprotección parental y los síntomas de estrés postraumático infantil. También se demostró una buena fiabilidad test-retest de la medida.

**Conclusiones**: El PTRQ es una evaluación autoaplicada válida y confiable de las cogniciones de los padres y del afrontamiento posterior al trauma del niño.

## Abstract (zh)

背景：在儿童遭受创伤之后，父母的反应在促进或阻碍儿童的心理调整方面发挥着重要作用。 然而，由于缺乏有效和可靠的测量方法，要考察创伤后期父母反应的作用不免受到阻碍。

目标：本研究的目的是设计并初步验证《父母创伤反应问卷（PTRQ）》，这是一项用于在儿童创伤后，父母自我报告对儿童应对状况的评估和支持的测量工具。

方法：我们对365名被试施测了一套有78题目的初始问卷，被试都是经历过创伤性事件的2-19岁的儿童的父母。 我们进行了主轴因子分析，然后通过与父母过度保护的标准化问卷对比评估减少题目后的问卷效度，以及与儿童创伤后心理健康的关联。

结果：因子分析产生了三个队父母适应不良评估的测量因素：（i）永久变化/损害，（ii）集中于儿童的脆弱性，以及（iii）自责。 另外，识别五个因素来评估父母对儿童应

对的支持：（i）行为逃避，（ii）认知回避，（iii）过度保护，（iv）保持创伤前日常，以及（v）趋近应对。 父母过度保护和儿童创伤后应激症状的测量验证了问卷具有良好的效度。 该问卷还显示出了的良好重测信度。

结论：PTRQ是在儿童创伤后的父母认知和应对的有效可靠的自评测量工具。

## 1. Introduction

Exposure to traumatic events is common in childhood and is associated with a range of adverse psychological outcomes, including post-traumatic stress disorder (PTSD) (Alisic et al., 2014; Hiller et al., 2016). One factor identified as a robust predictor of a child’s post-trauma psychological outcomes is their perceived social support (Kolaitis, 2017; Maercker & Hecker, 2016; Trickey, Siddaway, Meiser-Stedman, Serpell, & Field, 2012). Following a child’s experience of trauma, parents are often the primary source of support (Marsac, Donlon, Winston, & Kassam-Adams, 2013) and meta-analytic findings have confirmed that parenting style has a small yet significant impact on a child’s post-trauma mental health (Williamson, Creswell, Fearon et al., 2017). However, the extant literature has largely focused on general parenting responses, such as general overprotection, with little focus on parenting that may be trauma-specific, reflecting the lack of empirically validated, trauma-specific parenting measures.

In terms of the existing evidence base, parental overprotection and encouragement of avoidant coping (e.g., avoiding trauma reminders, discouraging trauma-related discussion) have previously been associated with poor child psychological adjustment following trauma (Bokszczanin, 2008; Ehlers, Mayou, & Bryant, 2003; Henry, Tolan, & Gorman-Smith, 2004). Based on cognitive models of PTSD (e.g., Ehlers & Clark, 2000), such coping styles have been hypothesized to increase children’s perception that they are very vulnerable or damaged by the event – a key predictor of poor psychological adjustment (Meiser-Stedman, Dalgleish, Glucksman, Yule, & Smith, 2009). Parental overprotection, including excessive involvement in a child’s activities and limited granting of autonomy, is also theorized to be a problematic parental response following child trauma. While it may be an understandable reaction to trying to prevent or reduce the child’s distress or chance of further harm (Scheeringa & Zeanah, 2001), this response can restrict children’s opportunities to engage with material or activities that may assist them in adaptively processing their trauma. Avoidant coping in the child is also likely to maintain fears (Marsac et al., 2016; Trickey et al., 2012). Conversely, providing opportunities for children to discuss the trauma, and positively reframe or confront trauma-related cues, may allow for negative appraisals to be addressed, anxiety responses to extinguish, and more complete trauma memories to be formed (Goodman, Rosenberg, Mueser, & Drake, 1997; Salmon & Reese, 2015). The broader child anxiety literature supports such potential for parents’ own maladaptive interpretations to influence anxiety in their child (e.g., Creswell, Shildrick, & Field, 2011). Similarly, there is evidence that parents can influence both the extent and content of child recall of negative events (Fivush, Hazzard, McDermott Sales, Sarfati, & Brown, 2003).

Another potential mechanism via which parents may have an input into child post-trauma adjustment is by providing adaptive coping assistance. Previous research has highlighted the importance of such positive parenting behaviours post-trauma, including supporting trauma-related discussions or modelling adaptive coping strategies (Alisic, Boeije, Jongmans, & Kleber, 2012; Marsac et al., 2013). However, evidence for a negative relationship between parental coping assistance and child post-traumatic stress symptom severity (PTSS) is mixed, with some studies finding weak or no evidence of an association (Punamaki et al., 2001;  Williamson, Creswell, Fearon et al., 2017). Evidence for the relationship between maladaptive parenting responses (e.g. overprotection, encouragement of avoidant coping) and child PTSS is more robust (e.g., Bokzczanin et al., 2008; Ehlers et al., 2003); nonetheless, our understanding of the relationship between parenting and child PTSS is hampered by the fact that many studies to date have utilized non-validated measures of post-trauma parenting responses.

Given the potentially important role of parental appraisals and behaviours in child adjustment, there is a need for valid, reliable assessments that measure parental responses following child trauma. Reliable assessment of parental post-trauma responses would delineate the relationship between child PTSD and parenting, and could identify parent-focused intervention targets. To address this, the aim of this study was to develop, and provide a preliminary validation of, a self-report parenting measure to be used in the aftermath of child trauma exposure. Specifically, the measure was designed to provide a tool that examines maladaptive post-trauma appraisals towards the child (e.g., the child being very vulnerable or permanently damaged) and the self (e.g., the parent being to blame), as well as how the parent supported the child’s coping. The support subscales focused on the type of coping that parents encouraged for the child, and included theoretically derived examples of adaptive coping (e.g., providing opportunities for the child to discuss the event) and maladaptive coping (e.g., discouraging conversation or avoiding reminders). We used factor analysis to refine the tool, combining data across five UK child trauma samples, to ensure a robust sample. To assess convergent validity, the resulting scale was compared to an existing measure of parental overprotection and to child PTSS, in a subsample of participants. Finally, we used intraclass correlation analyses to explore the test–retest reliability of the measure, using a subset of the total sample that had engaged a longitudinal design.

## 2. Method

This research received approval from the National Health Service Research Ethics Committee (Berkshire B 14/SC/0043; Cambridge South 12/EE/0458, 13/EE/0262; and Oxford A 13/SC/0599 committees), University of Reading Ethics Committee (UREC 14/20), the NRES Committee East of England (Cambridge South; 12/EE/0458), and the University of Bath Department of Psychology Research Ethics Committee (14-035; 15-218).

### 2.1. Participants

Participants were parents of trauma-exposed young people, drawn from five different samples, as detailed below. For all samples, exclusion criteria included: children with intellectual or developmental disability that precluded mainstream schooling; children with a history of organic brain damage; children currently presenting with self-harm behaviour or suicidal intent; caregiver’s or child’s inability to speak English; and where there were child protection concerns. Sample characteristics and event types are described in Table 1. Of the 365 participating parents, most were mothers. Just under half of the children were female, and the children’s ages ranged from 2 to 19 years old, with an average of 8 years old. More than half of the participants were recruited following a motor vehicle accident (MVA) or other accidental injury, but a diverse range of traumas were represented across the sample as a whole (including physical and sexual assaults and young people exposed to multiple traumas).

**Table 1.** Participant characteristics, reported by study sample.

Characteristic	PROTECT (*N* = 128)	PROTECT- Qual (*N* = 26)	PROSPECTS (*N* = 7)	PYCES (*N* = 127)	Online (*N* = 77)	Total (*N* = 365)
Age (years), *M* (*SD*)	9.8 (2.0)	10.6 (3.0)	13.3 (3.1)	5.7 (1.8)	8.5 (4.5)	8.2 (3.4)
Age range (years)	6–13	6–16	9–16	3–8	2–19	2–19
Proportion male	79 (61.7)	16 (61.5)	2 (28.9)	71 (55.9)	33 (42.9)	201 (55.1)
Proportion mothers	115 (89.8)	20 (76.9)	6 (85.7)	117 (92.1)	66 (90.4)	324 (89.8)^1^
Trauma type^2^						
Motor vehicle accident	61 (48.0)	10 (38.5)	0 (0)	19 (15.1)	7 (9.7)	97 (27.1)
Serious accidental injury	39 (30.7)	6 (23.0)	0 (0)	60 (47.6)	10 (13.9)	115 (32.2)
Acute medical episode	10 (7.9)	4 (15.4)	0 (0)	18 (14.3)	10 (13.9)	42 (11.7)
Burn	1 (0.8)	0 (0)	0 (0)	12 (9.5)	0 (0)	13 (3.6)
Non-sexual assault	2 (1.6)	2 (7.7)	0 (0)	6 (4.8)	4 (5.6)	14 (3.9)
Sexual assault	0 (0)	0 (0)	0 (0)	1 (0.8)	8 (11.1)	9 (2.5)
Multiple traumas	0 (0)	0 (0)	7 (100)	0 (0)	17 (23.6)	24 (6.7)
Other event	14 (11.0)	4 (15.4)	0 (0)	10 (7.9)	16 (22.2)	44 (12.3)

Data are shown as *n* (%) unless otherwise indicated.

^1^ Missing data for four cases from online sample;

^2^ missing data for six cases, one from PYCES, five from online sample.

### 2.2. Samples

Participants were derived from five different UK samples, resulting in a total of 365 participants (see Table 1 for study sample details). The studies were as follows.

#### 2.2.1. PROTECT

Children and their parents or guardians were enrolled in a study of parental responses to child experiences of trauma (Halligan, 2013). Families were recruited for the study via emergency departments and assessed approximately 1 month post-trauma, with follow-up data collected 3 months and 6 months later [including completion of the Parent Trauma Response Questionnaire (PTRQ) at each time-point]. Index traumas were primarily MVAs and other serious accidental injuries (e.g., serious falls).

#### 2.2.2. PYCES

Children and their parents or guardians participated in a randomized clinical trial of trauma-focused cognitive behaviour therapy (TF-CBT) (Dalgleish et al., 2015). Participants were recruited via emergency departments and other relevant sources [including child and adolescent mental health services (CAMHS), schools, victim support agencies, general practice surgeries and local newspapers]. Index traumas in this sample were predominantly accidental injuries. All participating children met Diagnostic and Statistical Manual of Mental Disorders, Fifth Edition (DSM-5) criteria for PTSD (American Psychiatric Association, 2013). The PTRQ was administered as part of a questionnaire battery, prior to the young person beginning treatment for their PTSD.

#### 2.2.3. Online

An online survey was used to recruit parents, advertised via schools, relevant online platforms and social media, with parents invited to participate if they had a child who had experienced a very frightening event. Index traumas were varied and included multiple traumas, physical and sexual assaults, as well as accidental injuries. (As this sample was collected online, as an initial sensitivity analysis the factor analyses were also run excluding these participants. The pattern of results was the same and the sample was thus retained in the final analyses.)

#### 2.2.4. PROTECT-Qual

Parents or guardians of children were recruited from emergency departments, CAMHS or the Child Bereavement, Trauma and Emotional Wellbeing Service (CHUMS), following child trauma exposure, as part of a qualitative study investigating parental responses post-trauma (Halligan, 2013; Williamson, Creswell, Butler, Christie, & Halligan, 2016; 2017). Index traumas in this sample were primarily MVAs and accidental injuries. The PTRQ was completed as part of a questionnaire battery either by post or online.

#### 2.2.5. PROSPECTS

A small sample of parents and children were derived from the PROSPECTS study (Meiser-Stedman et al., 2017). Children with a diagnosis of PTSD following exposure to multiple traumatic events were recruited as per the PYCES sample (see Subsection 2.2.2). Again, the PTRQ was completed as part of a questionnaire battery, prior to treatment.

### 2.3. Development of the PTRQ

The initial list of items was generated based on reviews of the qualitative and quantitative literature (e.g., Alisic et al., 2012; Fivush et al., 2003; Marsac et al., 2016; Scheeringa & Zeanah, 2001), cognitive and behavioural models of PTSD (e.g., Ehlers & Clark, 2000; Meiser-Stedman, 2002), existing measures of more general parenting (e.g., Edwards, Rapee, & Kennedy, 2010) and measures of adult post-trauma cognitions (Foa et al., 1999). The list was then subject to expert review. As the original item set (see supplementary material) was designed to be inclusive of a range of potential appraisals and support styles, this expert review was focused on consensus that appropriate items had been covered and that there was no unnecessary repetition. Pilot work with parents was used to explore the acceptability of the item wording.

#### 2.3.1. Item format

The initial item set involved 78 items, divided into two subscales: parental appraisals and parental support (see supplementary material). The appraisal scale consisted of 44 items rated on a four-point Likert scale (0 = Don’t agree at all, 1 = Agree slightly, 2 = Agree quite a lot, 3 = Agree completely). The support scale consisted of 34 items assessing how parents supported their child’s coping, 11 of which were cast in positive terms (e.g., ‘I have tried not to change my child’s usual routine since the event’), rated on a four-point Likert scale (0 = Not at all, 1 = A little, 2 = Some, 3 = A lot). Support items included responses that might be considered adaptive (e.g., providing opportunities to talk to the child about what happened) and maladaptive (e.g., encouraging the child to avoid reminders).

### 2.4. Validity measures

The PROTECT and PROTECT-Qual studies provided data that could be used to assess convergent and criterion validity.

#### 2.4.1. Parental overprotection

The 19-item Parental Overprotection Scale (POS) (Edwards et al., 2010) is a self-report measure that assesses general parental behaviours that restrict children’s exposure to situations perceived to be threatening or harmful. Items are rated on a five-point scale (0 = Not at all to 4 = Very much; scores range from 0 to 76). The POS was initially validated using a sample of parents of preschool children and has since been successfully used with parents of school-aged children (Clarke, Cooper, & Creswell, 2013). The good internal consistency of this measure was replicated (*α* = .92).

#### 2.4.2. Child PTSD symptom severity

The parent-report version of the University of California at Los Angeles (UCLA) Post-Traumatic Stress Disorder Reaction Index (PTSD-RI) (Pynoos, Rodriguez, Steinberg, Stuber, & Frederick, 1998) was used to assess child symptom severity. The PTSD-RI is a well-validated measure of PTSS, assessing 17 PTSD symptoms in the Diagnostic and Statistical Manual of Mental Disorders, 4th Edition, Text Revision (DSM-IV-TR) (American Psychiatric Association, 2000), each rated on a five-point Likert scale, ranging from 0 (‘None of the time’) to 4 (‘Most of the time’). The strong internal consistency of this measure was confirmed (*α* > .89).

### 2.5. Analytic plan

We used a comprehensive factor analytic strategy to reduce the number of items and explore the latent factor structure of the PTRQ across the five samples. *A priori*, it was decided to conduct factor analysis separately on the PTRQ appraisal and support scales in order to index each separately. An exploratory factor analysis using principal axis factoring (PAF) was conducted to define the underlying latent factors and generate factor scores (Fabrigar, Wegener, Maccallum, & Strahan, 1999; Widaman, 1993). PAF was chosen, rather than principal components analysis (PCA), as PAF estimates factor loadings and factor correlations more realistically than PCA as it recognizes the existence of random error introduced by measurement (Baglin, 2014), and is consequently less likely to produce inflated factor loadings or to underestimate factor correlations (Fabrigar et al., 1999). An oblique rotation was chosen for the PAF analysis as correlations between factors were anticipated. The number of factors to retain was based on several criteria: (1) a visual examination of the scree plot; (2) parallel analysis using the Monte-Carlo program; and (3) considerations regarding the meaning and interpretability of the factor model. Items that loaded more than .5 on a primary factor and less than .3 on remaining factors were retained (Matsunaga, 2011). Items that did not load more than .5 on a primary factor could be retained so long as these items had loadings of less than .3 on secondary factors and their inclusion improved the internal consistency of the subscales. Individual items were also assessed for face validity. The internal consistency of factors was examined using Cronbach’s alpha, with the threshold of .7 used to indicate acceptable reliability.

Once the subscales were finalized, we first explored whether the child’s age or gender was associated with parental responses on any subscale. Validity was then examined based on data provided by the PROTECT studies, using bivariate correlations to test associations. Convergent validity was based on examining correlations between the PTRQ factors and POS scores for PROTECT participants (*n *= 127). Criterion validity was examined by testing for correlations with child PTSS (UCLA) using combined data from the PROTECT and PROTECT-Qual studies (*n* = 154). In particularly stringent analyses, we also explored the usefulness of the measure above the standard measure of overprotection. Here, we used linear regression to explore whether the PTRQ subscales (all entered in a single step) predicted child PTSS, even after controlling for POS scores in Step 1. Finally, we examined test–retest reliability based on intraclass correlation analyses with absolute agreement, using data from the longitudinal PROTECT study (*n *= 127).

## 3. Results

### 3.1. Factor analysis and item retention

#### 3.1.1. Appraisal scale factor analysis

The appraisal PTRQ items were submitted to a PAF (Table 2). The Kaiser–Meyer–Olkin (KMO) measure confirmed the sampling adequacy for the analysis (KMO = .93) and Bartlett’s test of sphericity [*χ* ^2^(435) = 7039.69, *p *< .001] suggested that correlations between items were suitably large, confirming the appropriateness of the analysis. An examination of the scree plot and parallel analysis suggested that the appraisal subscale items best fit a three-factor model. From the initial 44 items, 14 were removed owing to poor factor loadings. Item 9 was retained, despite a primary factor loading of .4, as this item had a loading of less than .3 on secondary factors and its inclusion improved the internal consistency of the subscale. The PAF was then rerun with the final 30 items; the first factor explained 37.6% of the variance, with an additional 9.4% and 6.5% explained by the second and third factors, respectively. Examination of items loading on each factor suggests that the factors represent (i) permanent change, (ii) preoccupation with child’s vulnerability, and (iii) self-directed blame. Internal consistency was good across the three factors (permanent change: *α* = .91; vulnerability: *α* = .90; blame: *α* = .86).

**Table 2.** Principal axis factoring (PAF) factor loadings of the Parent Trauma Response Questionnaire (PTRQ) appraisals scale.

Item	Permanent change	Preoccupation–vulnerability	Blame
27. My child was so badly scared by the frightening event that they won’t get over it.	.86		
29. Our family will not get back to the way we were before the event happened.	.82		
28. Our family cannot recover from this sort of stress.	.82		
25. My child is always going to be anxious and upset now.	.78		
1. Our family will never be the same again.	.74		
12. My child is not going to be able to cope in the future now.	.71		
18. If my child has any more stress it will seriously damage him/her	.66		
38. Our family cannot cope very well with stress now.	.65		
5. My child has been permanently damaged by the frightening event.	.63		
17. My child would not be able to deal with being reminded of what happened.	.58		
42. I keep wishing we could have the life we had before the event happened.	.58		
9. My child might easily go to pieces if I don’t protect them from their fears.	.49		
41. I can’t bear to think about what happened to my child.		.77	
15. I get upset or angry when I am reminded of what happened to my child.		.76	
36. It is extremely upsetting to imagine how my child felt during the frightening event.		.74	
3. I keep thinking how it could have been even worse than it was.		.73	
40. I could not bear it if my child was ever hurt or threatened again.		.72	
26. I keep on wishing that I could go back in time and stop the event from happening.		.69	
14. I ask myself over and over why this happened to my child.		.69	
43. I can’t stop thinking about what could have been done to stop the event from happening.		.64	
37. I find it hard to control my feelings about happened to my child.		.56	
31. I am not going to risk my child being hurt again in the future.		.53	
39. Anything could happen to my child when I am not around.		.52	
44. Others must think I am a terrible parent.			.80
34. Others blame me for what happened to my child.			.78
22. I failed to look after my child properly.			.73
33. Others have judged me for what happened.			.69
32. I should have done more to keep my child safe.			.64
16. Others must wonder if I am safe looking after children.			.59
11. Another parent would not have let this happen			.59

Factor loadings less than .3 are suppressed.

#### 3.1.2. Support scale factor analysis

PAF was conducted on the support scale items (Table 3), with the KMO (.78) and Bartlett’s test [*χ* ^2^(190) = 2702.14, *p *< .001] confirming the acceptability of this analysis. Visual examination of the scree plot and parallel analysis indicated a five-factor model. Of the initial 34 items in the original PTRQ support scale, 14 were removed owing to poor factor loadings. Items 22 and 16 were retained, despite a primary factor loading of .4, as these items had loadings of less than .3 on secondary factors and their inclusion improved the internal consistency of the subscales. The PAF was rerun with these remaining items. The final 20-item coping scale was found to account for 50.9% of the total variance, with 19.2% of variance explained by the first factor, and 15.0%, 6.6%, 5.4% and 4.8% explained by the second, third, fourth and fifth factors, respectively. The five factors retained comprised subscales on: (i) overprotection, (ii) encouraging behavioural avoidance, (iii) maintaining normal, pre-trauma routines, (iv) encouraging approach coping, and (v) encouraging cognitive avoidance. The internal consistency of the coping factors ranged from adequate to good (overprotection *α* = .72; cognitive avoidance *α* = .90; behavioural avoidance *α* = .78; maintaining pre-trauma routines *α* = .68; encouraging approach coping *α* = .71).

**Table 3.** Principal axis factoring (PAF) factor loading of the Parent Trauma Response Questionnaire (PTRQ) support scale.

Item	Overprotection	Behavioural avoidance	Maintaining routines	Approach coping	Cognitive avoidance
18. Since the event I make sure I can always contact my child if s/he is not with me.	.78				
34. I need to know where my child is all the time, since the event happened.	.64				
12. I warn my child about possible dangers whenever I can.	.59				
30. I tell my child never to take any risks.	.55				
28. I plan with my child what they should do in an emergency.	.50				
22. I try to make my child understand that the world isn’t safe.	.41				
8. I avoid places, people or activities that might remind my child of what happened.		.92			
9. I try never to take my child near reminders of what happened.		.87			
6. I am careful about what we watch on the television and internet, so my child is not reminded of what happened.		.72			
20. I’ve tried not to change my child’s usual routine.			.85		
19. I try not to let my child’s possible fears or worries after the event change what we do.			.72		
26. I’ve tried to keep our lives as normal as possible since what happened.			.61		
16. Since the event, I try to get my child to do exactly the same things that they always did.			.48		
13. I’ve talked to my child about their feelings when they remember what happened.				.82	
7. I’ve talked to my child about how they felt at the time of the frightening event.				.71	
11. I’ll talk about what happened openly, even if my child is there.				.62	
23. I talk about the frightening event with my child just like I do anything else.				.52	
14. I tell my child not to think about what happened.					.86
15. I tell my child to put any thoughts or worries about what happened out of their head.					.80
3. If my child mentions what happened I try to distract them so they talk about something else instead.					.50

Factor loadings less than .3 are suppressed.

### 3.2. Child’s age and gender

Using data from the combined five samples, the child’s age was significantly associated with parent report on the maintaining ‘pre-trauma routines’ appraisals subscale (*p *= −.14, *p *= .01) and ‘overprotection’ support subscale (*r *= .18, *p *= .001), but not with any other subscale scores (all *r* < .08, *p* > .11). Higher endorsement of trying to return to pre-trauma routines and higher endorsement of overprotection were both associated with the child being younger. The gender of the child was not significantly associated with parent report on any of the PTRQ subscales (all *rs* < .10, *ps* > .14).

### 3.3. Convergent and criterion validity

Convergent validity of the PTRQ was assessed against a standardized measure of general parent overprotection (POS). The correlation matrix is presented in Table 4. The total score on the POS was significantly associated with all PTRQ subscales, with the exception of the maintaining pre-trauma routines and encouraging approach coping subscales (i.e. the two putatively ‘adaptive’ coping subscales). The same pattern of results was found when criterion validity was assessed using parent-reported child PTSS. That is, higher child symptom scores were significantly associated with higher endorsement of maladaptive appraisals and coping, but not with the ‘adaptive’ coping subscales (Table 4).

**Table 4.** Bivariate correlations between the Parent Trauma Response Questionnaire (PTRQ) subscales and the Parental Overprotection Scale (POS).

	1.	2.	3.	4.	5.	6.	7.	8.	9.	10.	11.
**1. PTRQ appraisals scale total**											
2. PTRQ – permanent change	.83**										
3. PTRQ – preoccupation–vulnerability	.91**	.58**									
4. PTRQ – blame	.72**	.43**	.56**								
**5. PTRQ support scale total^1^**	.45**	.30**	.53**	.18**							
6. PTRQ – maintaining routines	−.03	−.14**	.08	−.08	.55**						
7. PTRQ – approach coping	−.13*	−.21**	−.02	−.12*	.46**	.33**					
8. PTRQ – behavioural avoidance	.62**	.63**	.51**	.35**	.42**	−.08	−.19**				
9. PTRQ – cognitive avoidance	.35**	.34**	.33**	.14**	.44**	.07	.16**	.38**			
10. PTRQ – overprotection	.50**	.36**	.56**	.24**	.77**	.13*	.08	.33**	.31**		
11. POS	.61**	.54**	.58**	.42**	.60**	.10	.02	.57**	.36**	.65**	
12. PTSD reaction index (parent report)	.55**	.51**	.48**	.32**	.19*	−.09	−.10	.41**	.26**	.18*	.38**

^1^Maintaining pre-trauma routines and approach coping subscales were reversed in calculating the total score.

PTSD, post-traumatic stress disorder.

*p < .05; **p < .001.

As both POS parental overprotection scores and PTRQ scales were each associated with child PTSD symptoms (Table 4), we further explored whether PTRQ scores provided extra predictive power above the shorter POS. Multiple regression analysis demonstrated that the PTRQ subscales predicted a significant amount of variance in parent-reported child PTSS (*R^2^* change = .43, *F* = 13.2, df = 8109, *p* < .001), even after controlling for POS scores in Step 1 (*R* ^2^ *= .12, F* = 19.6, df = 1117, *p* < .001). In the final model, PTRQ subscales of preoccupation with vulnerability (*b *= .45, *t *= 3.56, *p = *.001), permanent change (*b *= .26, *t *= 2.54, *p = *.013) and behavioural avoidance (*b *= .21, *t *= 2.18, *p = *.031) each explained unique variance, whereas POS score was no longer a significant predictor (*b *= −.03, *t *= −.32, *p = *.75).

### 3.4. Test–retest reliability

We used intraclass correlation coefficients (ICCs), with absolute agreement, to assess test–retest reliability. Data were from the longitudinal PROTECT study based on assessments at 1 month post-trauma and follow-ups 3 months and 6 months later (*n *= 127). Data were analysed for agreement between subscale scores at 1 month and 3 months, and again between scores at 3 months and 6 months. All three of the maladaptive appraisal subscales’ ICC values were above .75, representing excellent reliability and suggesting that scores remained relatively stable between time-points [permanent change: 1–3 months (1–3mo) *r*_ic_ = .86, 3–6 months (3–6mo) *r*_ic_ = .92; vulnerability: 1–3mo *r*_ic_ = .89, 3–6mo *r*_ic_ = .91; blame: 1–3mo *r*_ic_ = .87, 3–6mo *r*_ic_ = .86]. From the support subscales, the ICC value was excellent for overprotection (1–3mo *r*_ic_ = .81, 3–6mo *r*_ic_ = .82), good for cognitive avoidance (1–3mo *r*_ic_ = .70, 3–6mo *r*_ic_ = .75) and behavioural avoidance (1–3mo *r*_ic_ = .67, 3–6mo *r*_ic_ = .73), and fair for maintaining pre-trauma routines (1–3mo *r*_ic_ = .61, 3–6mo *r*_ic_ = .60) and approach coping (1–3mo *r*_ic_ = .46, 3–6mo *r*_ic_ = .52).

## 4. Discussion

A lack of empirically validated measures has hindered robust conclusions about the role of parental responses following child trauma exposure. Here, we developed and conducted an initial validation of the PTRQ as a measure of parental appraisals and support style following a child’s experience of trauma. These preliminary results indicate that the PTRQ is a valid and reliable measure of parental responses following child trauma exposure, with subscales covering parental appraisals about the child and themselves, potentially maladaptive elements of support and potentially adaptive support.

A 30-item scale of parental maladaptive appraisals was supported by factor analysis, within which two subscales explored appraisals related to the child or family ((i) permanent change or damage, and (ii) preoccupation with child’s vulnerability) and one related to appraisals about the parent themselves (i.e. that they were to blame). All three subscales showed good internal consistency, and correlated with a standard parental overprotection measure and with the parent report of the child’s PTSS. The processes by which parental appraisals may have an impact on child outcomes remain an important area for future research, with the broader child anxiety literature suggesting that the pathway may be through the impact on the child’s own appraisals or interpretation of the event (Creswell, Schniering, & Rapee, 2005; Dadds, Barrett, Rapee, & Ryan, 1996; Fivush, 2007). Consistent with this hypothesis, preliminary evidence suggests that child appraisals mediate an association between general perceived social support and child PTSS (Hiller et al., 2017; Hitchcock, Ellis, Williamson, & Nixon, 2015).

Analysis of PTRQ parental support items generated three subscales regarding maladaptive elements of support and two focusing on putatively adaptive support [maladaptive: (i) encouraging behavioural avoidance, (ii) encouraging cognitive avoidance, and (iii) general overprotection; and adaptive: (i) encouraging approach coping, and (ii) maintaining pre-trauma routines]. Again, all subscales demonstrated good internal consistency. Although the cognitive and behavioural avoidance subscales consisted of only three items each, both had good internal consistency and all of the corresponding items loaded above .6, suggesting that both are stable factors (Guadagnoli & Velicer, 1988; Osborne & Costello, 2009). We found that maladaptive support components, including trauma-specific maladaptive support (i.e. endorsing avoidance of trauma cues) and general overprotective support style, were associated with both a standard measure of parental overprotection and higher child PTSS. The association between maladaptive parenting style and child PTSS is consistent with preliminary empirical evidence that parents’ own coping and response can influence a child’s post-trauma coping (Cobham, McDermott, Haslam, & Sanders, 2016; Marsac et al., 2013) and psychological adjustment (Ostrowski, Christopher, & Delahanty, 2006). Such parenting behaviours may increase a child’s perceived vulnerability to threat post-trauma and prevent the elaboration and processing of the child’s trauma memory (Salmond et al., 2011; Wood et al., 2003). That the trauma-specific support component (i.e. endorsing avoidance) predicted child PTSS, even above any association between standard overprotection and PTSS, demonstrates the usefulness of assessing trauma-specific parental behaviours, as enabled by the PTRQ. In contrast, the two ‘adaptive’ support subscales were not associated with either parental general overprotection or child PTSS. Although still acceptable, these subscales showed slightly lower internal consistencies and poorer test–retest reliabilities than other scales, suggesting that they may be more complex domains to index.

There is some evidence that either too much or too little trauma-related talk may predict child distress in post-disaster samples (Cobham et al., 2016), again consistent with complexity in providing positive support. The logical conclusion based on our findings in relation to maladaptive support is that addressing parental tendencies to support avoidant coping in the child could be beneficial. As such, when considering implications for intervention design, it is particularly important to know that parental support for approach coping (i.e. facing reminders of the trauma) was not associated with harm. At the same time, it is worth noting that parents who were particularly low on support for trauma talk and approach coping (i.e. were particularly avoidant of conversations around the trauma) may have been unlikely to participate in the PROTECT study, on which validity analyses were primarily based, as it required parents and children to talk about trauma material (Hiller et al., 2017). The role of adaptive parenting behaviours and coping assistance remains underresearched in the trauma field (Williamson, Creswell, Fearon et al., 2017), yet parents often primarily report increases in warmth and emotional support for their child following acute trauma (Alisic et al., 2012; Williamson et al., 2016). Future research on the potential role of adaptive parenting remains warranted.

Child developmental factors are also potentially relevant when examining parental responses following child trauma exposure. For example, it may be entirely appropriate for parents of younger children to report needing to know their child’s whereabouts at all times (e.g., PTRQ item 34). Equally, some of the cognition-focused questions may also be less relevant for parents of young children (e.g., encouraging them not to think about the event). In the current study, we found surprisingly little evidence that the age of the child was associated with the parent report on the PTRQ subscales. The only exception to this was the child’s age being inversely associated with parent reports of both overprotection and trying to return to pre-trauma routines. These findings may reflect differences in parenting different-aged children, with parents potentially having more control over, and responsibility for, the activities of younger children than older children or teens. That said, as most other subscales (e.g., ratings of vulnerability, avoidant coping) were not significantly associated with the child’s age, it is difficult to draw definitive conclusions on the role of the child’s age in relation to parental post-trauma responses. The PTRQ was ultimately designed for use with parents of school-aged children and older (> 5 years old) and further research is required to explore the suitability of this measure, or an adapted version, for use with parents of pre-schoolers.

Examination of the impact of the parent’s own trauma history, distress or PTSS on their PTRQ responses was beyond the scope of this preliminary validation study. As parental PTSS has been found to impact not only post-trauma parenting behaviours but also child PTSD outcomes (e.g., Leen-Feldner et al., 2013), this should be considered in future investigations. Similarly, examination of how parental responses on the PTRQ relate to actual parenting behaviours was also beyond the scope of the present study. However, research by Hiller et al. (2017) has found preliminary evidence that the PTRQ subscales capture actual parental responses. Specifically, when parents were observed discussing the trauma with their child in an observational task, more frequent expression of negative interpretations (e.g. emphasis on the child being very vulnerable or damaged by the event) was positively associated with PTRQ scales indexing perceptions of child damage and vulnerability, and cognitive and behavioural avoidance promotion, and inversely associated with endorsement of approach coping (see Hiller et al., 2017, supplementary material). There was less consistent evidence that parental emphasis on avoidant coping observed in the same narrative task related to PTRQ scores. Nonetheless, the presence of significant associations between observed parenting and responses on the PTRQ further supports the validity of the PTRQ as a reliable measure of post-trauma parental responses (Hiller et al., 2017).

Evidence that parents’ scores on the PTRQ are associated with their behaviour towards the child and with child PTSS suggests that the tool could have clinical utility. For example, the PTRQ could be used to identify families with particularly negative or anxious parenting practices and facilitate the provision of support or guidance for behavioural change (Cobham et al., 2016). That said, whether child symptoms evoke negative parenting or *vice versa* remains unclear; it is possible that effective treatment of child PTSS alone may lead to changes in parenting practices (King et al., 2000; Silverman et al., 2008). Overall, these implications underscore the need for validated and reliable measures, such as the PTRQ, to allow for greater exploration of parenting after trauma, in terms of both mechanism research and implications for treatment outcomes.

The results of this study should be considered in light of its limitations. First, the associations between maladaptive parental appraisals and support and child PTSS provide preliminary support for the validity of the PTRQ. As parent report was used for both parenting and child PTSS this may have introduced potential single-reporter bias. However, Hiller et al. (2017) found that the PTRQ subscales correlated significantly with child report of PTSS, including longitudinally. Additional research is necessary to confirm these observations and allow causal inferences to be drawn about the impact of parenting behaviours on child post-trauma adjustment. Secondly, as is typical in parenting research, the majority of participating caregivers were mothers and future studies should include more fathers to gain a deeper understanding of post-trauma parental responses. Study samples were also relatively culturally homogeneous, and primarily consisted of young people who had experienced accidental injury (e.g., MVA). Non-English-speaking families and families where there was concern over child maltreatment/protection were not included in any of the studies, so findings cannot necessarily be generalized to these groups. In addition, data for the initial validity analyses and test–retest reliability against the overprotection measure and parent-reported child PTSS were only available for a subset of school-aged participants, where most young people had experienced a single-incident accidental trauma. Thus, the conclusions that can be made about the generalizability of these findings to preschool age children or older adolescents is limited. Of note, for the factor analysis component, participating families were recruited from several publicly funded healthcare centres; thus, these samples are likely to reflect demographic characteristics of the local community and be representative of children receiving healthcare treatment in the UK. Nonetheless, we were unable to explore the role of broader demographic factors, such as socio-economic status, in relation to PTRQ scores, as this was not routinely available across studies. It is also notable that when PTRQ scales were correlated with other parenting measures (indexing observed trauma-related parenting and parental overprotection), the correlations were somewhat non-specific, with associations between seemingly less similar appraisal and behavioural indices being present alongside associations that might arguably be expected to be stronger (e.g., between two overprotection measures). This could reflect overlapping and mutually influential processes (e.g., between parental appraisals and behaviours) but may also be a function of shared underlying factors (e.g., parental anxiety). Finally, while the sample size was relatively small, with a variable ratio of 5:1, in general a sample size of over 200 is considered fair for an initial validation (Comrey & Lee, 1992). Nevertheless, further replication with a larger sample is needed. That our sample size was produced as a result of combining data across five separate studies demonstrates the challenges with recruiting trauma-exposed child samples, and the importance of research groups exploring options to share data to allow for necessarily larger samples.

In sum, this preliminary validation of the PTRQ found that this measure is a reliable and valid assessment of parental appraisals and support style following child trauma exposure. The PTRQ captures a range of parental appraisals regarding themselves and the child, as well as a range of adaptive and maladaptive support strategies post-trauma. The use of the PTRQ in studies of risk and protective factors following child trauma exposure will allow for a more robust exploration of the role of parenting in the aftermath of trauma.
