# Supplementary material for: The Parent Trauma Response Questionnaire (PTRQ): development and preliminary validation
Source: Eur J Psychotraumatol. 2018 Jun 20;9(1):1478583. doi: 10.1080/20008198.2018.1478583 (PMC6008584; doi:10.1080/20008198.2018.1478583)
Supplement: Supplementary_Material_PTRQ.docx [file ZEPT_A_1478583_SM1090.docx]

**Supplementary Material (S1): List of original items.**

The following questions are about thoughts that some parents have after their child has had a very frightening experience. Please indicate how much you agree with each one.

|  |  | *Don’t agree at all* | *Agree slightly* | *Agree quite a lot* | *Agree completely* |
| --- | --- | --- | --- | --- | --- |
| 1 | Our family will never be the same again. | [ ] | [ ] | [ ] | [ ] |
| 2 | I have to make sure I can protect my child all the time. | [ ] | [ ] | [ ] | [ ] |
| 3 | I keep thinking how it could have been even worse than it was. | [ ] | [ ] | [ ] | [ ] |
| 4 | What happened has changed the way that people see our family for the worse. | [ ] | [ ] | [ ] | [ ] |
| 5 | My child has been permanently damaged by the frightening event. | [ ] | [ ] | [ ] | [ ] |
| 6 | I blame myself for what happened. | [ ] | [ ] | [ ] | [ ] |
| 7 | What happened to my child is down to me as a parent. | [ ] | [ ] | [ ] | [ ] |
| 8 | My child has been emotionally scarred by the frightening event. | [ ] | [ ] | [ ] | [ ] |
| 9 | My child might easily go to pieces if I don’t protect them from their fears. | [ ] | [ ] | [ ] | [ ] |
| 10 | Our family has been disrupted really badly by what happened. | [ ] | [ ] | [ ] | [ ] |
| 11 | Another parent would not have let this happen. | [ ] | [ ] | [ ] | [ ] |
| 12 | My child is not going to be able to cope in the future now. | [ ] | [ ] | [ ] | [ ] |
| 13 | My child is not safe when they are away from me. | [ ] | [ ] | [ ] | [ ] |
| 14 | I ask myself over and over why this happened to my child. | [ ] | [ ] | [ ] | [ ] |
| 15 | I get upset or angry when I am reminded of what happened to my child. | [ ] | [ ] | [ ] | [ ] |
| 16 | Others must wonder if I am safe looking after children. | [ ] | [ ] | [ ] | [ ] |
| 17 | My child would not be able to deal with being reminded of what happened. | [ ] | [ ] | [ ] | [ ] |
| 18 | If my child has any more stress it will seriously damage him/her | [ ] | [ ] | [ ] | [ ] |
| 19 | My child is not tough enough to cope with things that can happen. | [ ] | [ ] | [ ] | [ ] |
| 20 | I keep thinking again and again “If only this hadn’t happened to us.” | [ ] | [ ] | [ ] | [ ] |
| 21 | The world is too dangerous for my child. | [ ] | [ ] | [ ] | [ ] |
| 22 | I failed to look after my child properly. | [ ] | [ ] | [ ] | [ ] |
| 23 | My child could be hurt by anyone. | [ ] | [ ] | [ ] | [ ] |
| 24 | Good parents keep an eye on their children 100% of the time. | [ ] | [ ] | [ ] | [ ] |
| 25 | My child is always going to be anxious and upset now. | [ ] | [ ] | [ ] | [ ] |
| 26 | I keep on wishing that I could go back in time and stop the event from happening. | [ ] | [ ] | [ ] | [ ] |
| 27 | My child was so badly scared by the frightening event that they won’t get over it. | [ ] | [ ] | [ ] | [ ] |
| 28 | Our family cannot recover from this sort of stress. | [ ] | [ ] | [ ] | [ ] |
| 29 | Our family will not get back to the way we were before the event happened. | [ ] | [ ] | [ ] | [ ] |
| 30 | I cannot trust anyone else to look after my child. | [ ] | [ ] | [ ] | [ ] |
| 31 | I am not going to risk my child being hurt again in the future. | [ ] | [ ] | [ ] | [ ] |
| 32 | I should have done more to keep my child safe. | [ ] | [ ] | [ ] | [ ] |
| 33 | Others have judged me for what happened. | [ ] | [ ] | [ ] | [ ] |
| 34 | Others blame me for what happened to my child. | [ ] | [ ] | [ ] | [ ] |
| 35 | It’s completely up to me to make sure that my child is safe. | [ ] | [ ] | [ ] | [ ] |
| 36 | It is extremely upsetting to imagine how my child felt during the frightening event. | [ ] | [ ] | [ ] | [ ] |
| 37 | I find it hard to control my feelings about what happened to my child. | [ ] | [ ] | [ ] | [ ] |
| 38 | Our family cannot cope very well with stress now. | [ ] | [ ] | [ ] | [ ] |
| 39 | Anything could happen to my child when I am not around. | [ ] | [ ] | [ ] | [ ] |
| 40 | I could not bear it if my child was ever hurt or threatened again. | [ ] | [ ] | [ ] | [ ] |
| 41 | I can’t bear to think about what happened to my child. | [ ] | [ ] | [ ] | [ ] |
| 42 | I keep wishing we could have the life we had before the event happened. | [ ] | [ ] | [ ] | [ ] |
| 43 | I can’t stop thinking about what could have been done to stop the event from happening. | [ ] | [ ] | [ ] | [ ] |
| 44 | Others must think I am a terrible parent. | [ ] | [ ] | [ ] | [ ] |

The following questions are about the things that some parents describe doing after their child has had a very frightening experience. Please indicate how much you have done each one. There are no right or wrong answers.

|  |  | *Not at all* | *A little* | *Some* | *A lot* |
| --- | --- | --- | --- | --- | --- |
| 1 | I avoid talking about the event because I don’t want to upset my child. | [ ] | [ ] | [ ] | [ ] |
| 2 | I take extra care to make sure that our family is safe. | [ ] | [ ] | [ ] | [ ] |
| 3 | If my child mentions what happened I try to distract them so they talk about something else instead. | [ ] | [ ] | [ ] | [ ] |
| 4 | I try to keep conversations away from what happened in the event. | [ ] | [ ] | [ ] | [ ] |
| 5 | I check every place we visit now, to make sure that there is nothing dangerous. | [ ] | [ ] | [ ] | [ ] |
| 6 | I am careful about what we watch on the television and internet, so my child is not reminded of what happened. | [ ] | [ ] | [ ] | [ ] |
| 7 | I’ve talked to my child about how they felt at the time of the frightening event. | [ ] | [ ] | [ ] | [ ] |
| 8 | I avoid places, people or activities that might remind my child of what happened. | [ ] | [ ] | [ ] | [ ] |
| 9 | I try never to take my child near reminders of what happened. | [ ] | [ ] | [ ] | [ ] |
| 10 | I don’t let my child do anything that might be risky now. | [ ] | [ ] | [ ] | [ ] |
| 11 | I’ll talk about what happened openly, even if my child is there. | [ ] | [ ] | [ ] | [ ] |
| 12 | I warn my child about possible dangers whenever I can. | [ ] | [ ] | [ ] | [ ] |
| 13 | I’ve talked to my child about their feelings when they remember what happened. | [ ] | [ ] | [ ] | [ ] |
| 14 | I tell my child not to think about what happened. | [ ] | [ ] | [ ] | [ ] |
| 15 | I tell my child to put any thoughts or worries about what happened out of their head. | [ ] | [ ] | [ ] | [ ] |
| 16 | Since the event, I try to get my child to do exactly the same things that they always did. | [ ] | [ ] | [ ] | [ ] |
| 17 | If my child brings up what happened then I make sure I spend some time talking about it with them. | [ ] | [ ] | [ ] | [ ] |
| 18 | Since the event I make sure I can always contact my child if s/he is not with me. | [ ] | [ ] | [ ] | [ ] |
| 19 | I try not to let my child’s possible fears or worries after the event change what we do. | [ ] | [ ] | [ ] | [ ] |
| 20 | I’ve tried not to change my child’s usual routine. | [ ] | [ ] | [ ] | [ ] |
| 21 | When someone in my family mentions the event, I tell them to stop bringing it up. | [ ] | [ ] | [ ] | [ ] |
| 22 | I try to make my child understand that the world isn’t safe. | [ ] | [ ] | [ ] | [ ] |
| 23 | I talk about the frightening event with my child just like I do anything else. | [ ] | [ ] | [ ] | [ ] |
| 24 | I try to stop other people talking about what happened in front of my child. | [ ] | [ ] | [ ] | [ ] |
| 25 | I’ve taken my child places that are likely to remind them of what happened. | [ ] | [ ] | [ ] | [ ] |
| 26 | I’ve tried to keep our lives as normal as possible since what happened. | [ ] | [ ] | [ ] | [ ] |
| 27 | Since the event I have stopped my child from going some places that they used to go to. | [ ] | [ ] | [ ] | [ ] |
| 28 | I plan with my child what they should do in an emergency. | [ ] | [ ] | [ ] | [ ] |
| 29 | I don’t discuss what happened in front of my child. | [ ] | [ ] | [ ] | [ ] |
| 30 | I tell my child never to take any risks. | [ ] | [ ] | [ ] | [ ] |
| 31 | I have stopped my child from doing certain things so that he/she is not reminded of what happened. | [ ] | [ ] | [ ] | [ ] |
| 32 | I answer any questions my child has about what happened as fully as I can. | [ ] | [ ] | [ ] | [ ] |
| 33. | I tell my child not to trust anyone. | [ ] | [ ] | [ ] | [ ] |
| 34 | I need to know where my child is all the time, since the event happened. | [ ] | [ ] | [ ] | [ ] |

**Supplementary Material (S2). Final version of the PTRQ**

**Parent Trauma Response Questionnaire (PTRQ)**

The following questions are about thoughts that some parents have after their child has had a very frightening experience. Please indicate how much you agree with each one.

|  |  | *Don’t agree at all* | *Agree slightly* | *Agree quite a lot* | *Agree completely* |
| --- | --- | --- | --- | --- | --- |
| 1 | Our family will never be the same again. | [ ] | [ ] | [ ] | [ ] |
| 2 | I keep thinking how it could have been even worse than it was. | [ ] | [ ] | [ ] | [ ] |
| 3 | My child has been permanently damaged by the frightening event. | [ ] | [ ] | [ ] | [ ] |
| 4 | My child might easily go to pieces if I don’t protect them from their fears. | [ ] | [ ] | [ ] | [ ] |
| 5 | Another parent would not have let this happen. | [ ] | [ ] | [ ] | [ ] |
| 6 | My child is not going to be able to cope in the future now. | [ ] | [ ] | [ ] | [ ] |
| 7 | I ask myself over and over why this happened to my child. | [ ] | [ ] | [ ] | [ ] |
| 8 | I get upset or angry when I am reminded of what happened to my child. | [ ] | [ ] | [ ] | [ ] |
| 9 | Others must wonder if I am safe looking after children. | [ ] | [ ] | [ ] | [ ] |
| 10 | My child would not be able to deal with being reminded of what happened. | [ ] | [ ] | [ ] | [ ] |
| 11 | If my child has any more stress it will seriously damage him/her | [ ] | [ ] | [ ] | [ ] |
| 12 | I failed to look after my child properly. | [ ] | [ ] | [ ] | [ ] |
| 13 | My child is always going to be anxious and upset now. | [ ] | [ ] | [ ] | [ ] |
| 14 | I keep on wishing that I could go back in time and stop the event from happening. | [ ] | [ ] | [ ] | [ ] |
| 15 | My child was so badly scared by the frightening event that they won’t get over it. | [ ] | [ ] | [ ] | [ ] |
| 16 | Our family cannot recover from this sort of stress. | [ ] | [ ] | [ ] | [ ] |
| 17 | Our family will not get back to the way we were before the event happened. | [ ] | [ ] | [ ] | [ ] |
| 18 | I am not going to risk my child being hurt again in the future. | [ ] | [ ] | [ ] | [ ] |
| 19 | I should have done more to keep my child safe. | [ ] | [ ] | [ ] | [ ] |
| 20 | Others have judged me for what happened. | [ ] | [ ] | [ ] | [ ] |
| 21 | Others blame me for what happened to my child. | [ ] | [ ] | [ ] | [ ] |
| 22 | It is extremely upsetting to imagine how my child felt during the frightening event. | [ ] | [ ] | [ ] | [ ] |
| 23 | I find it hard to control my feelings about what happened to my child. | [ ] | [ ] | [ ] | [ ] |
| 24 | Our family cannot cope very well with stress now. | [ ] | [ ] | [ ] | [ ] |
| 25 | Anything could happen to my child when I am not around. | [ ] | [ ] | [ ] | [ ] |
| 26 | I could not bear it if my child was ever hurt or threatened again. | [ ] | [ ] | [ ] | [ ] |
| 27 | I can’t bear to think about what happened to my child. | [ ] | [ ] | [ ] | [ ] |
| 28 | I keep wishing we could have the life we had before the event happened. | [ ] | [ ] | [ ] | [ ] |
| 29 | I can’t stop thinking about what could have been done to stop the event from happening. | [ ] | [ ] | [ ] | [ ] |
| 30 | Others must think I am a terrible parent. | [ ] | [ ] | [ ] | [ ] |

The following questions are about the things that some parents describe doing after their child has had a very frightening experience. Please indicate how much you have done each one. There are no right or wrong answers.

|  |  | *Not at all* | *A little* | *Some* | *A lot* |
| --- | --- | --- | --- | --- | --- |
| 1 | If my child mentions what happened I try to distract them so they talk about something else instead. | [ ] | [ ] | [ ] | [ ] |
| 2 | I am careful about what we watch on the television and internet, so my child is not reminded of what happened. | [ ] | [ ] | [ ] | [ ] |
| 3 | I’ve talked to my child about how they felt at the time of the frightening event. | [ ] | [ ] | [ ] | [ ] |
| 4 | I avoid places, people or activities that might remind my child of what happened. | [ ] | [ ] | [ ] | [ ] |
| 5 | I try never to take my child near reminders of what happened. | [ ] | [ ] | [ ] | [ ] |
| 6 | I’ll talk about what happened openly, even if my child is there. | [ ] | [ ] | [ ] | [ ] |
| 7 | I warn my child about possible dangers whenever I can. | [ ] | [ ] | [ ] | [ ] |
| 8 | I’ve talked to my child about their feelings when they remember what happened. | [ ] | [ ] | [ ] | [ ] |
| 9 | I tell my child not to think about what happened. | [ ] | [ ] | [ ] | [ ] |
| 10 | I tell my child to put any thoughts or worries about what happened out of their head. | [ ] | [ ] | [ ] | [ ] |
| 11 | Since the event, I try to get my child to do exactly the same things that they always did. | [ ] | [ ] | [ ] | [ ] |
| 12 | Since the event I make sure I can always contact my child if s/he is not with me. | [ ] | [ ] | [ ] | [ ] |
| 13 | I try not to let my child’s possible fears or worries after the event change what we do. | [ ] | [ ] | [ ] | [ ] |
| 14 | I’ve tried not to change my child’s usual routine. | [ ] | [ ] | [ ] | [ ] |
| 15 | I try to make my child understand that the world isn’t safe. | [ ] | [ ] | [ ] | [ ] |
| 16 | I talk about the frightening event with my child just like I do anything else. | [ ] | [ ] | [ ] | [ ] |
| 17 | I’ve tried to keep our lives as normal as possible since what happened. | [ ] | [ ] | [ ] | [ ] |
| 18 | I plan with my child what they should do in an emergency. | [ ] | [ ] | [ ] | [ ] |
| 19 | I tell my child never to take any risks. | [ ] | [ ] | [ ] | [ ] |
| 20 | I need to know where my child is all the time, since the event happened. | [ ] | [ ] | [ ] | [ ] |
